# Management of PFO in paradoxical embolic stroke with hemorrhagic conversion: a case report

**DOI:** 10.3389/fcvm.2024.1395542

**Published:** 2024-09-18

**Authors:** Michael Sabina, Aqeel Khanani, Joshua Tsai, Amanda Rigdon, Joseph Massaro

**Affiliations:** Graduate Medical Education, Internal Medicine, Lakeland Regional Health Medical Center, Lakeland, FL, United States

**Keywords:** paradoxical embolism, patent foramen ovale, deep venous thrombosis, middle cerebral artery stroke, hemorrhagic conversion, inferior vena cava (IVC) filter, PFO closure

## Abstract

A paradoxical embolism is defined as a venous thrombus that crosses through a heart defect, into the systemic circulation, usually through a patent foramen ovale. Treatment varies between closure of patent foramen ovale vs. medical management based on a variety of individual risk factors and the cardiac defect's characteristics. We describe a case of paradoxical stroke complicated by hemorrhagic conversion, ultimately requiring an IVC filter.

## Introduction

Globally, stroke is the second highest cause of death and ranks fifth in the United States. In the U.S., the middle cerebral artery (MCA) is impacted in over half of all stroke cases (1). Each year, about 18,000 patients aged 18–60 in the United States will present with a PFO and a cryptogenic stroke (2). Immediate treatment of confirmed stroke cases is crucial, employing intravenous tissue plasminogen activator (TPA) within 4.5 h of symptom onset, or thrombectomy within 24 h (3). Afterwards, identifying the thrombus source is essential, typically cardiac in nature. However, in some instances there is a venous origin from the deep veins of the leg that traverses into systemic circulation via a cardiac defect, known as a paradoxical embolism (4). This report discusses a left MCA stroke patient, presumed to have a deep vein thrombosis (DVT) traversing through a patent foramen ovale (PFO) into cerebral circulation, and our unique management approach in the context of hemorrhagic conversion and hemodynamic instability.

## Case presentation

**Figure 1:**
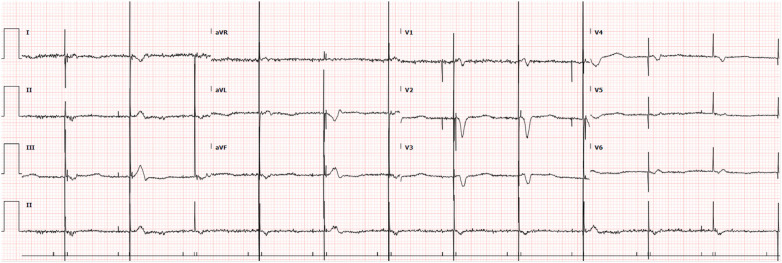
Electrocardiogram showing atrial and ventricular paced rhythm.

**Figure 2:**
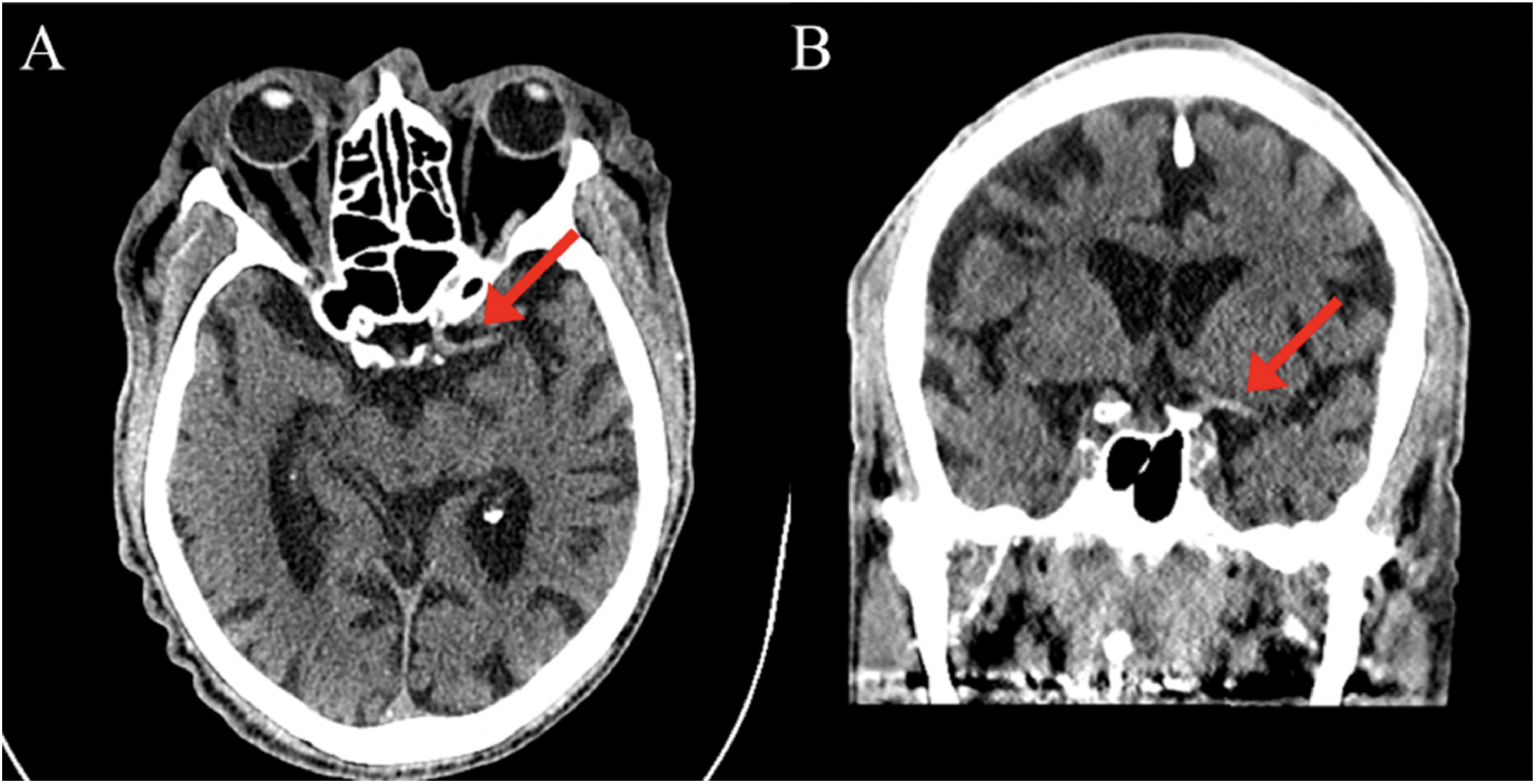
CT head: Hyperdense M1 segment of middle cerebral artery (red arrows) on axial view **(A)** and coronal view **(B)**.

**Figure 3:**
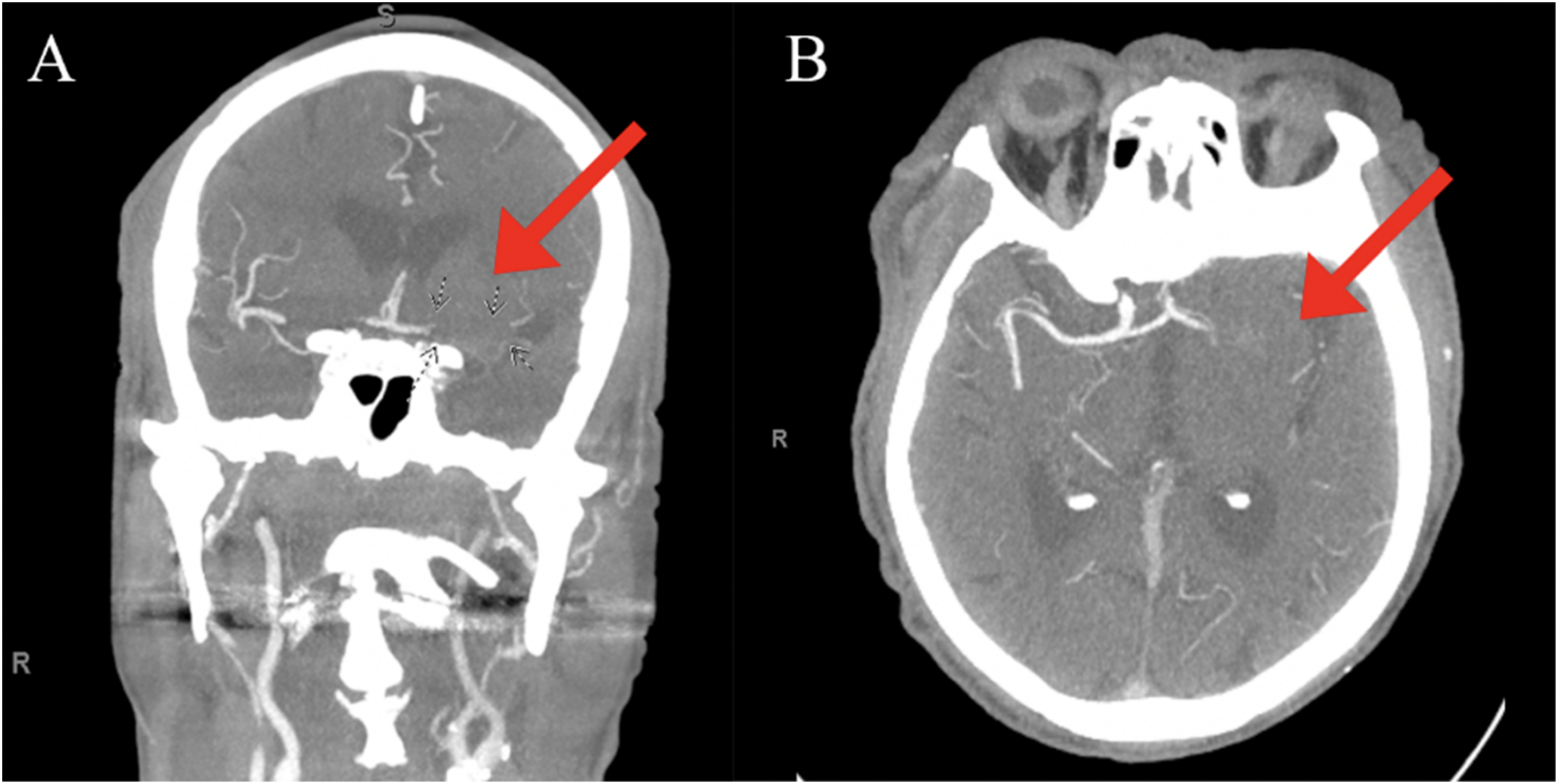
CT angiogram head/neck: Subtotal occlusion of the M1 segment of left middle cerebral artery (red arrows) with globally decreased visualization of the M2 and M3 segment vessels on coronal **(A)** and axial **(B)** views.

**Figure 4:**
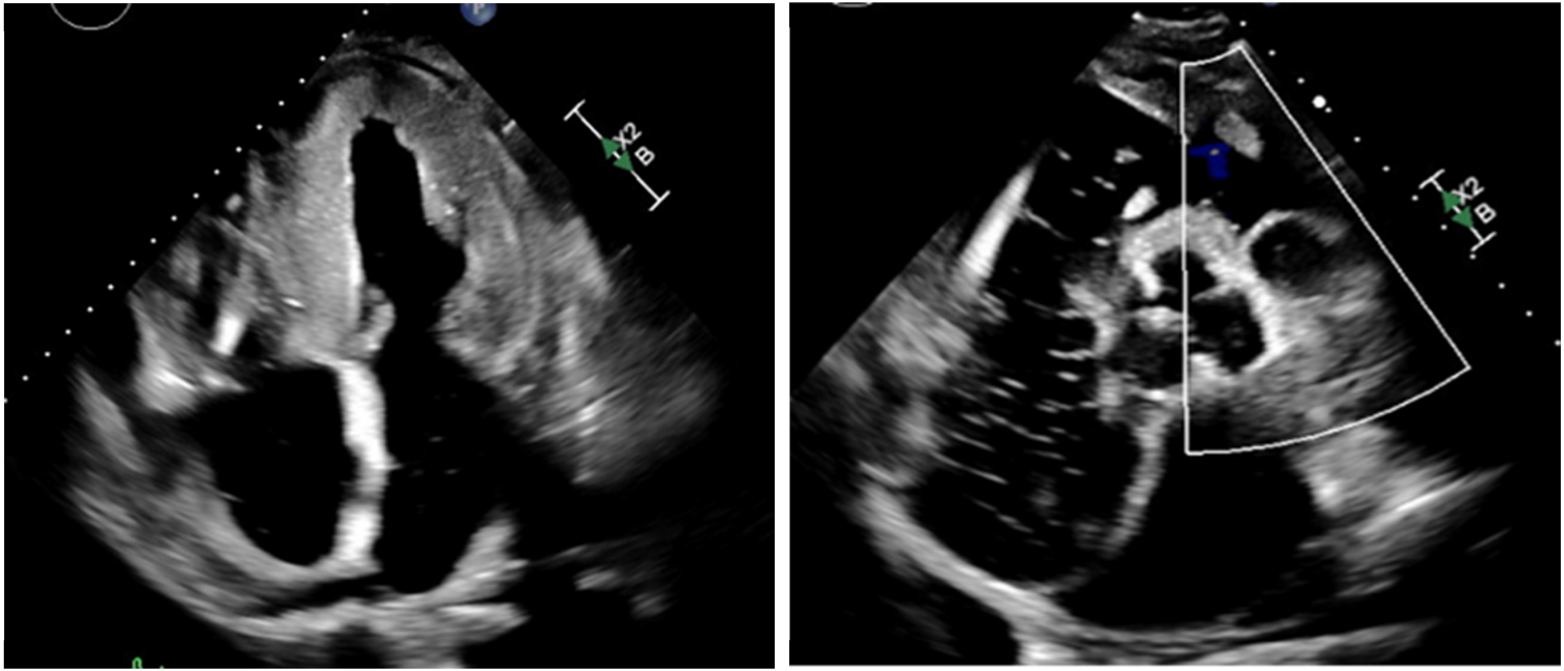
Transthoracic echocardiogram with apical four chamber view with and without Doppler; echogenic small bubbles in left atrium and left ventricle. Notable patent foramen ovale vs. atrial septal defect.

**Figure 5:**
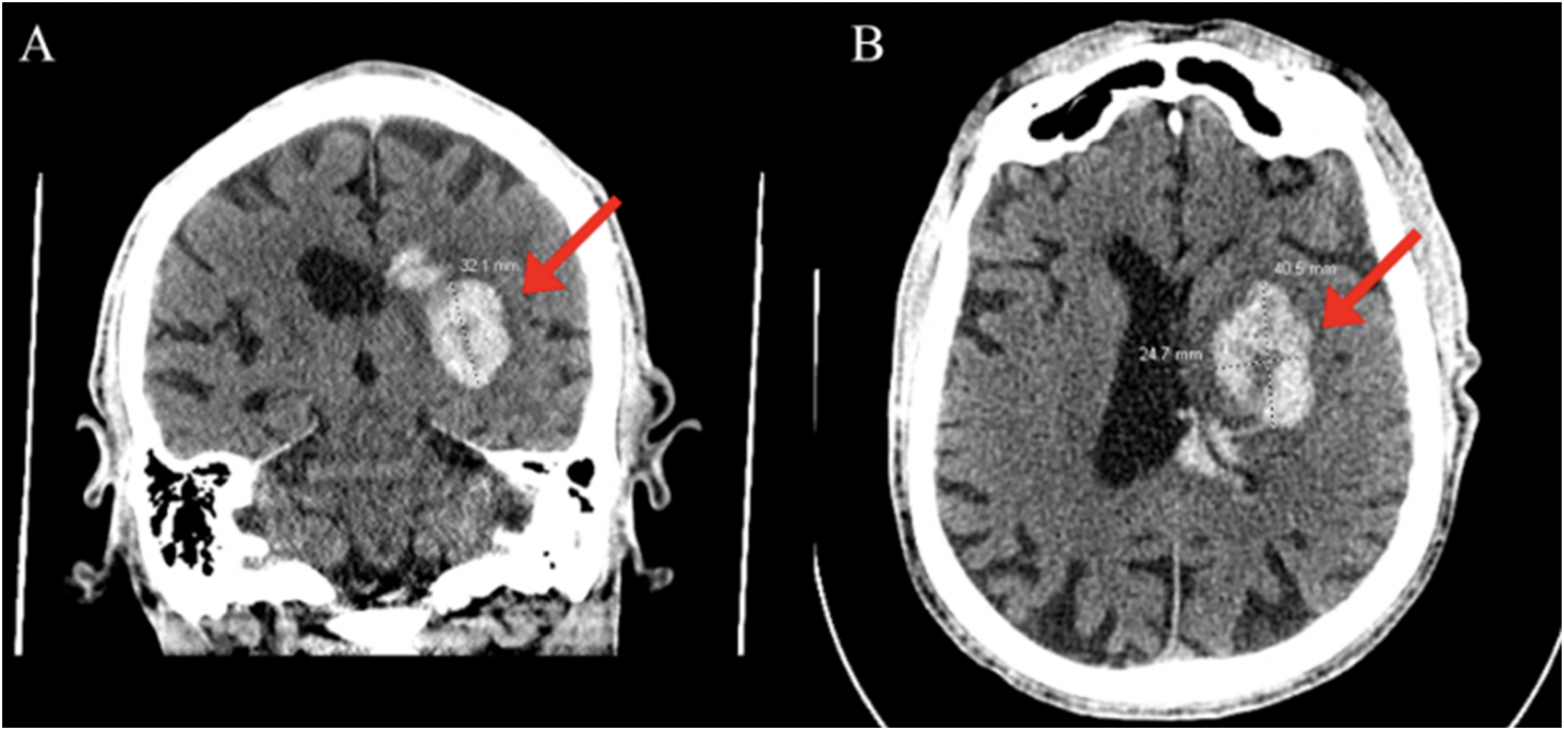
Large intraparenchymal hemorrhage at the level of the left basal ganglia (red arrows) with intraventricular extension and mass effect on the left lateral ventricle, no midline shift. Coronal **(A)** and axial **(B)** views.

An 83-year-old male with a past medical history of atrial fibrillation with atrial and ventricular pacemaker, heart failure with improved ejection fraction with ICD, prior myocardial infarction not treated with percutaneous coronary intervention (PCI) and stenting, prior transient ischemic attacks, and diabetes, presented with right-sided facial droop, right upper and lower extremity weakness, and aphasia with a last known normal of 15 h prior. Vitals on arrival were only pertinent for uncontrolled blood pressure of 142/79 mmHg, heart rate of 70 beats per minute, 16 breaths per minute, and 98% oxygen saturation on room air. Home medications included Rivaroxaban 20 mg daily, Amiodarone 200 mg daily, Sacubitril-Valsartan 24–26 mg twice a day, Carvedilol 3.125 mg twice a day, Dapagliflozin 10 mg daily, and Atorvastatin 40 mg daily, all orally administered. Initial laboratory tests (Table 1) revealed elevated high-sensitivity troponins with a notable delta, alongside elevated pro-BNP levels, suggesting myocardial strain or ischemia. There was also notably elevated thyroid stimulating hormone (TSH), suggesting a likely untreated underlying hypothyroidism. Mild thrombocytopenia was present on admission. The electrocardiogram, taken while transporting the patient for an emergent computed tomography (CT) scan of the head, displayed an atrial and ventricular paced rhythm (Figure 1), so we were unable to assess for arrythmias or ischemic changes accurately. The initial non-contrast CT of the head (Figure 2) identified a hyperdense M1 segment in the left middle cerebral artery (MCA), indicative of a MCA stroke. Subsequent CT angiography of the head and neck (Figure 3) revealed a near-total occlusion of the M1 segment of the left MCA and significantly reduced visibility of the M2 and M3 segment vessels, further supporting a stroke diagnosis. A cerebral angiogram was performed with a successful M1 thrombectomy. Antiplatelet therapy was deferred as anticoagulation therapy would be initiated in the future for atrial fibrillation. However, due to recency of stroke, we held anticoagulation on day one. Due to concerns regarding the thrombus's origin, further diagnostic tests were conducted. A transthoracic echocardiogram (TTE) displaying echogenic bubbles (Figure 4), suggesting a patent foramen ovale (PFO), but no signs of cardiac thrombus. We questioned the family members at bedside if they were aware of any heart defects during prior cardiac workups given his TIA and MI history, but they were unsure. Patient was aphasic and unable to provide information. A transesophageal echocardiogram (TEE) was to be done the following day of admission to definitively rule out a cardiac thrombus and assess more adequately the size and shunt of the PFO. Given these suspected cardiac findings, a venous etiology for the cerebrovascular event was considered, prompting a venous ultrasound of the lower extremities. This ultrasound revealed multiple deep vein thrombosis in the left popliteal, posterior tibial, and peroneal veins. A follow-up CT scan on day two of the patient's admission showed hemorrhagic conversion of the left MCA stroke, characterized by extensive intraparenchymal hemorrhage at the left basal ganglia level (Figure 5). Given the patient's hemodynamic instability and the risk of exacerbating the intracranial hemorrhage, we opted against transcatheter closure of PFO and instead chose to implant an inferior vena cava (IVC) filter. The patient unfortunately was unable to recover, and a decision was made to proceed with hospice care where the patient expired shortly after.

**Table 1:** Initial labs on admission.

Labs on admission		
Parameters	Patient values	Reference range
WBC	3.27 × 10^3^/µl	4.5–11.0 × 10^3^/µl
Hgb	13.4 g/dl	13.5–17.5 g/dl
Hct	37.4%	38.3–48.6%
Platelet	125 × 10^3^/µl ml/min/1.73 m^2^	150–450 × 10^3^/µl
eGFR	50 ml/min/1.73 m^2^	>90 ml/min/1.73 m^2^
Glucose	155 mg/dl	70–99 mg/dl (fasting)
Creatinine	1.39 mg/dl	0.74–1.35 mg/dl
Sodium	133 mEq/L	135–145 mEq/L
Potassium	4.7 mEq/L	3.5–5.0 mEq/L
Chloride	101 mEq/L	98–106 mEq/L
CO2	19 mEq/L	22–28 mEq/L
A1C	5.8%	<5.7%
Lactic acid	1.8 mmol/L	0.5–2.2 mmol/L
PT	17.8 s	9.5–13.5 s
INR	1.6	0.8–1.1
APTT	35.3 s	30–40 s
Pro-BNP	2,700 pg/ml	<125 pg/ml
HS troponin-T	145 ng/L	0–14 ng/L
HS troponin 1 h	123 ng/L	0–14 ng/L
HS troponin 6 h	127 ng/L	0–14 ng/L
1 h delta	−22	
6 h delta	−18	
T3 free	1.54 pg/ml	2.0–4.4 pg/ml
T4 free	1.39 ng/dl	0.82–1.77 ng/dl
TSH	19.3 µIU/ml	0.45–4.5 µIU/ml
Triglycerides	72 mg/dl	<150 mg/dl
Cholesterol	83 mg/dl	<200 mg/dl
HDL	45.6 mg/dl	>40 mg/dl
LDL	23 mg/dl	<100 mg/dl

## Discussion

In evaluating embolic strokes with a concurrent patent foramen ovale (PFO), it's crucial to identify the stroke's origin before labeling it as paradoxical. Key exclusions include atrial fibrillation and structural cardiac anomalies like atrial septal aneurysms, atrial septal defects, pulmonary arteriovenous malformations, lesions, tumors, or vegetations (3). Our case involved a patient with a history of atrial fibrillation and a dual-chamber pacemaker, complicating the assessment of atrial fibrillation or irregular activity that could facilitate clot formation. A transthoracic echocardiogram (TTE) eliminated potential sources such as vegetations or structural defects, leaving the PFO as the likely cause. Given the patient's hemodynamic instability and the risk of exacerbating the intracranial hemorrhage, we opted against PFO closure and instead chose to implant an inferior vena cava (IVC) filter. This strategy aims to mitigate the risk of recurrent paradoxical cerebral embolism in contexts where traditional anticoagulation is contraindicated and PFO closure is not ideal.

Generally, the management of PFO should align with the PFO-associated stroke causal likelihood (PASCAL) classification, which includes the Risk of Paradoxical Embolism (RoPE) score to inform decisions about PFO closure (5, 6). Current data on PFO management is mixed, and lackluster in the elderly population. The CLOSURE-1 trial found that PFO closure with a device did not offer greater benefit than medical therapy alone for preventing recurrent stroke or TIA in patients with cryptogenic stroke or TIA (7). Similarly, a 2013 trial showed that PFO closure for secondary prevention of cryptogenic embolism did not significantly reduce the risk of recurrent embolic events or death compared with medical therapy (8). Trials such as RESPECT LTF and CLOSE have shown lower rates of recurrent strokes with PFO closure in patients under 60 years (9, 10). However, data on elderly populations is lacking. The DEFENSE-PFO trial, although favorable, included patients aged 18–80 with a mean age of only 50 years and a small sample size (11). Ongoing research, including the CLOSE-2 and COACH ESUS trials, aims to clarify the role of PFO closure in older patients (12, 13). Currently, observational studies suggest transcatheter PFO closure can be safe and effective in older patients, but these decisions should be made on a case-by-case basis (14).

Understanding the mechanisms behind hemorrhagic transformation (HT) of ischemic stroke is crucial for preventing this severe complication. HT is influenced by various individual risk factors. A history of hypertension and acute hypertension at stroke onset are significant risk factors due to their impact on blood-brain barrier (BBB) disruption (15). Similarly, hyperglycemia at stroke onset is associated with worse outcomes and higher HT rates due to its effects on BBB permeability and oxidative stress (16). However, correcting serum glucose levels in acute stroke is debated; overcorrection may be fatal, as seen in the SHINE trial, which showed no significant benefit from intensive glucose control (17). Higher body weight, reflected by increased cardiovascular and metabolic stress, also correlates with an increased risk of symptomatic HT (18). Coagulation status, including INR levels, antiplatelet usage, and platelet count, plays a crucial role. Elevated INR and antiplatelet therapy can compromise hemostasis, increasing the risk of HT, especially following thrombolytic treatment (19).

Some risk factors for HT are unmodifiable, such as age, which brings structural changes in vasculature like increased rigidity and decreased elasticity, predisposing individuals to HT. Genetic factors, such as polymorphisms in MMP-9 and collagen IV, alter the structural integrity of blood vessels, increasing susceptibility to HT (20). The anatomical variability in collateral blood supply also affects HT likelihood, where better collateral circulation can reduce the ischemic core size and improve outcomes. The etiology of the occlusion and the side of the occluded vessel further influence HT risk, with cardioembolic strokes exhibiting different HT dynamics compared to strokes from large artery atherosclerosis. The severity of the initial stroke, as measured by clinical scales like the NIHSS, correlates with the extent of ischemic damage and the likelihood of subsequent HT, with more severe strokes generally indicating larger infarct sizes and higher HT risk (21).

By considering these diverse mechanisms and individual risk factors, clinicians can better stratify patients for thrombolytic therapy and other interventions, aiming to minimize the risk of HT and improve overall stroke outcomes (22).

## Conclusion

In paradoxical stroke, anticoagulation remains the mainstay of treatment when closure is not recommended. In circumstances where both closure and anticoagulation are not ideal, we report the use of an IVC filter as a strategic decision to reduce the risk of further venous embolic events while also minimizing the risk of exacerbating the current intracranial hemorrhage.

## Data Availability

The original contributions presented in the study are included in the article/Supplementary Material, further inquiries can be directed to the corresponding author.
